# Immune Cells in the Normal Ovary and Spontaneous Ovarian Tumors in the Laying Hen (*Gallus domesticus*) Model of Human Ovarian Cancer

**DOI:** 10.1371/journal.pone.0074147

**Published:** 2013-09-09

**Authors:** Michael J. Bradaric, Krishna Penumatsa, Animesh Barua, Seby L. Edassery, Yi Yu, Jacques S. Abramowicz, Janice M. Bahr, Judith L. Luborsky

**Affiliations:** 1 Department of Pharmacology, Rush University Medical Center, Chicago, Illinois, United States of America; 2 Department of Pathology, Rush University Medical Center, Chicago, Illinois, United States of America; 3 Department of Obstetrics & Gynecology, Rush University Medical Center, Chicago, Illinois, United States of America; 4 Department of Animal Science, University of Illinois Urbana-Champaign, Illinois, United States of America; University of Alabama at Birmingham, United States of America

## Abstract

**Background:**

Spontaneous ovarian cancer in chickens resembles human tumors both histologically and biochemically. The goal was to determine if there are differences in lymphocyte content between normal ovaries and ovarian tumors in chickens as a basis for further studies to understand the role of immunity in human ovarian cancer progression.

**Methods:**

Hens were selected using grey scale and color Doppler ultrasound to determine if they had normal or tumor morphology. Cells were isolated from ovaries (n = 6 hens) and lymphocyte numbers were determined by flow cytometry using antibodies to avian CD4 and CD8 T and B (Bu1a) cells. Ovarian sections from another set of hens (n = 26) were assessed to verify tumor type and stage and to count CD4, CD8 and Bu1a immunostained cells by morphometric analysis.

**Results:**

T and B cells were more numerous in ovarian tumors than in normal ovaries by flow cytometry and immunohistochemistry. There were less CD4+ cells than CD8+ and Bu1a+ cells in normal ovaries or ovarian tumors. CD8+ cells were the dominant T cell sub-type in both ovarian stroma and in ovarian follicles compared to CD4+ cells. Bu1a+ cells were consistently found in the stroma of normal ovaries and ovarian tumors but were not associated with follicles. The number of immune cells was highest in late stage serous tumors compared to endometrioid and mucinous tumors.

**Conclusions:**

The results suggest that similar to human ovarian cancer there are comparatively more immune cells in chicken ovarian tumors than in normal ovaries, and the highest immune cell content occurs in serous tumors. Thus, this study establishes a foundation for further study of tumor immune responses in a spontaneous model of ovarian cancer which will facilitate studies of the role of immunity in early ovarian cancer progression and use of the hen in pre-clinical vaccine trials.

## Background

Multiple elements are involved in the development and progression of cancer including genetic, epigenetic, environmental and immune factors [1], [2]. Although it is clear that immunity has a major role in cancer and that controlling immune responses to tumors has significant potential for cancer prevention and treatment, the immune response to tumors is not well understood. A higher tumor content of CD3+ T cells [3] or CD8+ cytotoxic T cells [4] in late stage tumors is associated with a better prognosis for ovarian cancer patients while a higher relative content of T regulatory cells is associated with a poorer prognosis [5], suggesting the number and types of immune cells are important for clinical outcomes. Recent evidence suggests that CD20+ B cells are found in both early and late stage ovarian tumors and that higher numbers may be related to better five year survival rates [6]. However there is conflicting data regarding the role of immunity in tumor prevention or progression and it has been suggested that the functional role of immunity changes during tumor progression [7].

Ovarian cancer is usually diagnosed in advanced stages and has a high rate of recurrence and mortality since there are no standard early detection methods. Because early stage ovarian cancer is difficult to detect, most studies use late stage specimens and thus there is relatively little information on immunity in the initiation and early progression of ovarian cancer. The early stages of ovarian cancer are more readily studied in animal models and these models represent an alternative approach to elucidating tumor etiology and the role of immunity in ovarian cancer. Further development of pre-clinical models of ovarian cancer is needed to facilitate development and testing of vaccines to treat ovarian cancer.

There are a number of rodent models of ovarian cancer based on genetically engineered or chemically induced tumors or on implantation of human tumors in SCID (Severe Combined Immunodeficiency) or RAG (Recombination activating gene) deficient mice [8]. However, most rodent models do not develop ovarian cancer spontaneously and those that do often produce only one histotype [8], [9], [10], [11], [12]. While these models are useful for insights into genetic and environmental factors contributing to cancers and to development of chemo-therapeutic strategies, they are less appropriate for investigation of early spontaneous events related to tumor immunology because it is not clear if they undergo the same natural or spontaneous events that lead to ovarian tumors.

The laying hen (*Gallus domesticus*) fills this gap since it spontaneously develops progressive ovarian tumors with later stage metastases and production of ascites [13]. We and others reported that the laying hen is a valid model for the study of human ovarian cancer [14], [15], [16], [17], [18]. Spontaneous ovarian tumors in the hen share many features of human ovarian cancer. The histology and morphology of hen and human ovarian tumors are similar; hen tumors commonly have serous, endometrioid, clear cell and mucinous histology comparable to the frequent epithelial subtypes of human ovarian cancer [14]. We showed previously that ovarian tumors in laying hens can also be detected by transvaginal ultrasound using the type of equipment commonly used in clinics [19], [20]. When combined with contrast agents [21], we were able to advance the stage at which ovarian tumor angiogenesis and small microscopic tumor foci were detected. Thus non-invasive methods for selecting hens for studies and for monitoring tumor progression are available. Furthermore, naturally occurring genetic mutations such as p53, RAS and Her2/neu are found in hen tumors [22], [23], [24]. Likewise, there is a growing list of proteins expressed in chicken ovarian tumors such as CA125 [25], mesothelin [26], COX 1 [27], [28], Selenium Binding Protein 1 [29], E-cadherin [30] and VEGF that are similarly altered in human ovarian tumors [17], [20], [31]. It is also striking that aspirin [32] and exogenous progesterone [33] partially reduce ovarian tumors similar to humans. Ovarian cancer in the hen has many of the facets of the human disease and therefore is a valid model.

The laying hen model is particularly appropriate for studies of ovarian cancer and immunity. The hen is well known for seminal contributions to our understanding of immunology including the first evidence for different lineages for T and B lymphocytes [34]. In the ovary, there is an increase in follicle associated T cells, macrophages and B cells during ovarian maturation and then a decrease with aging [35], [36], [37], [38]. In addition, the serum of hens with ovarian tumors contains anti-ovarian and anti-tumor antibodies [39] similar to women [40], [41], [42]. Thus, the laying hen model represents a feasible model to study immunity and early stage ovarian cancer.

However, the types and content of immune cells in hens with ovarian tumors and the changes compared to normal ovaries are unknown. Therefore, the goal of this cross-sectional study was to describe and quantify the T and B cells associated with ovarian tumors in the laying hen in order to establish a basis for studies of mechanisms of immunity in human ovarian cancer.

## Materials and Methods

### Animal Care and Selection

White leghorn hens (3 years old, strain W96) were housed at the University of Illinois at Urbana-Champaign poultry farm in the Department of Animal Science. Food and water were provided *ad libitum* and hens were maintained on a 17∶7 hours (light: dark) schedule. Ovarian morphology and angiogenesis were evaluated using transvaginal ultrasound scanning as described previously [19] and the data were used to select hens with normal ovaries or ovarian tumors. For flow cytometry, cells from the entire ovary were prepared without further histological evaluation. For immunohistochemical studies, hens were similarly selected. Normal or tumor histology and tumor stage were verified and tumor type was determined using Hematoxylin and Eosin (H&E) stained sections of ovary as described previously [14]. This study was carried out in accordance with the recommendations in the Guide for the Care and Use of Laboratory Animals of the National Institutes of Health. The protocol was approved by the Institutional Animal Care and Use Committees at Rush University Medical Center (number 08-011) and the University of Illinois (number 05147).

### Flow Cytometry

Flow cytometry was used to phenotype lymphocytes in hen ovaries (n = 6) and spleen (n = 2). T cells were detected with CD4-FITC and CD8-PE (Southern Biotech, Birmingham, AL) and B cells were detected with anti-Bu1a (Abcam, Cambridge, MA) conjugated to allophycocyanin (APC) (Zenon labeling system, Invitrogen, Carlsbad CA) according to the manufacturer’s instructions. The ovarian lymphocytes were detected using single-step flow cytometry [43]. Positive controls consisted of lymphocytes from spleens and negative controls included substitution of antibodies of the same isotype for primary antibodies (data not shown). The total number of immune cells was then calculated from the flow cytometry data and the total cell counts were obtained from the tissue extract.

Whole ovaries were cut into 2–3 cm pieces and cells were released by enzymatic digestion using collagenase (2,000 IU/1 gm of tissue; Worthington, Lakewood, NJ), DNase I (200 µg; Stemcell Technologies, Vancouver, BC), and DMEM/F12 media (Invitrogen, Carlsbad, CA) (40°C, 2 hours with shaking). The tubes were centrifuged (100×g, 5 minutes). The cell pellet was suspended in cold phosphate buffered saline (PBS) containing 1 mM EDTA (1 mL, 2 minutes), centrifuged (100×g; 5 minutes) and the pellet suspended in 10 mL Hank’s Balanced Salt Solution (HBSS) containing 2% fetal bovine serum (FBS). Cells were counted with a Coulter counter set for 5–11 microns. Cells (6×10^6^) were labeled and fixed in 2% paraformaldehyde and 5×10^5^ cells/sample were analyzed using a FACS Calibur (BD Biosciences, San Jose, CA). Data was analyzed with CellQuest 1 software (BD Biosciences, San Jose, CA).

### Tissue Preparation for Histology and Immunohistochemistry

Ovaries were fixed in 10% PBS-buffered formalin and embedded in paraffin. H&E stained sections of ovary were analyzed by a board-certified pathologist at Rush University for tumor type and stage based on similarities to human morphology as described previously [14].

A portion of each ovary was also embedded in OCT compound (Tissue Tek, Sakura, Torrence, CA), snap frozen in dry ice-cooled methanol and stored at −80°C. Twenty-four hours prior to sectioning on a Leicha cryostat, tissues were brought to −20°C and adhered to a metal stage with OCT embedding compound. Tissues were sectioned (10 µm), fixed in cold acetone (4°C, 20 minutes), air-dried at room temperature (30 minutes) and stored at −80°C until use.

### Immunohistochemistry

Immune cells were detected with antibodies against markers for hen CD4 T cells, CD8 T cells (Southern Biotech, Birmingham, AL) and B cells (Bu1a/chB6) (Abcam, Cambridge, MA). Sections were immunostained using a kit with diaminobenzidine (DAB) as a substrate (Vector Labs, Burlingame, CA). Sections were washed (15 minutes), counterstained with Hematoxylin (Fisher Scientific, Rockford, IL), dehydrated, applied to slides and dried (37°C, 16 hours). Microscopy was performed on a Nikon Microphot FXA microscope.

### Morphometric Analysis of Immune Cells

Ovarian sections were stained with antibodies against CD4, CD8, and Bu1a and were examined using a light microscope (Olympus BX-41; Olympus America Inc., Center Valley, PA). Immunopositive cells were counted using MicroSuite Five software (Olympus America Inc.). Briefly, a minimum of three sections from each ovary from twenty-six hens was counted per immune cell marker (CD4, CD8 and Bu1a) for a total of 243 slides (3 slides×27 ovaries×3 markers).

Total immune cells were counted in each section. For each section, 5 to 20 random fields were counted at a magnification of 20x (or 84,000 µm^2^) depending on the size of the specimen. Cell counts were averaged for each section to normalize the data since a different number of fields were counted in different size sections. Three sections were averaged for every hen for each marker. The values for each group (normal, early, and late tumors) were averaged and then expressed as the average number of cells in 8.4×10^4^ µm^2^ of ovarian tissue. Group designations (normal, early and late stage cancer) were based on histological assessment of H&E stained sections following previously defined staging for hen ovarian cancer [14].

In addition, the number of cells in follicles of the same sections was counted and expressed as the number of immune cells per 2×10^5^ µm^2^ of follicle area. Immune cells in atretic (dying) follicles [44] were avoided since they are known to contain an influx of lymphocytes presumably in response to apoptosis [45], [46].

### Statistical Analyses

Statistical tests were performed using SPSS (Chicago, IL). The Mann Whitney U test was used to determine if mean differences were significant with p<0.5 considered significant.

## Results

### Hen Selection and Ovarian Morphology

**Figure 1:**
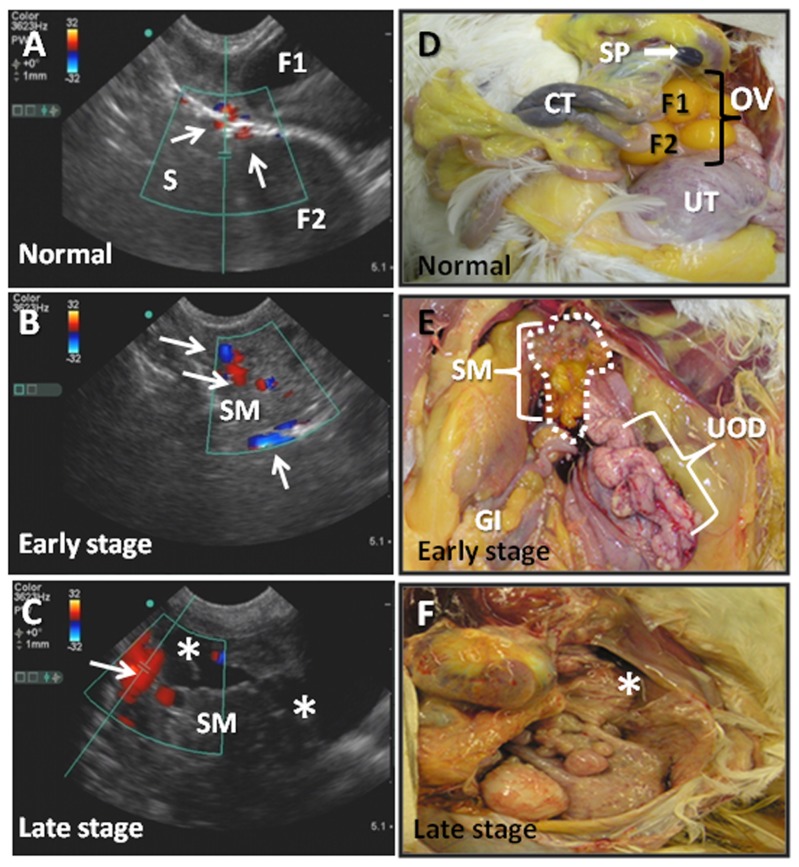
Selection of hens with normal ovaries or ovarian tumors using (A–C) color Doppler ultrasound and (D–F) gross morphology of the ovaries. A normal ovary (**A and D**) with maturing ovarian follicles (F1, F2) and stromal blood vessels (arrows) with normal gross morphology showing maturing (F1, F2) ovarian follicles. An example of an abnormal ovary (**B and E**) that contains more blood vessels (arrows) than in the normal ovary, no detectable large mature follicles and a small solid tissue mass (circle) in the ovary. An example of late stage ovarian cancer (**C**) characterized by a large solid mass with increased blood flow (arrows) and profuse ascites (*).The gross morphology (**F**) confirmed the presence of multiple solid masses and tumor metastasis to other organs. Abbreviations: CT = cecal tonsil; F1–F2 = large preovulatory follicles; GI = gastrointestinal tract; OV = ovary; S = ovarian stroma; SM = solid tissue mass in the ovary; SP = spleen; UOD = upper oviduct; UT = uterus.

Hens were selected into groups with and without ovarian tumors based on color Doppler ultrasound and gross morphology of the ovary. As described previously, normal ovaries contain multiple developing follicles (**Figure 1A and 1D**) and discrete blood vessels (**Figure 1A**) in the stroma surrounding ovarian follicles. In early stage ovarian cancer, the ovary contains fewer developing follicles, and more blood vessels than in normal ovaries (**Figure 1B and 1E**). In late stage ovarian cancer, a solid mass with ascites is evident and there are no developing follicles (**Figure 1C and 1F**).

### Localization of Lymphocytes in Normal Ovary, Early Stage and Late Stage Ovarian Tumors

**Figure 2:**
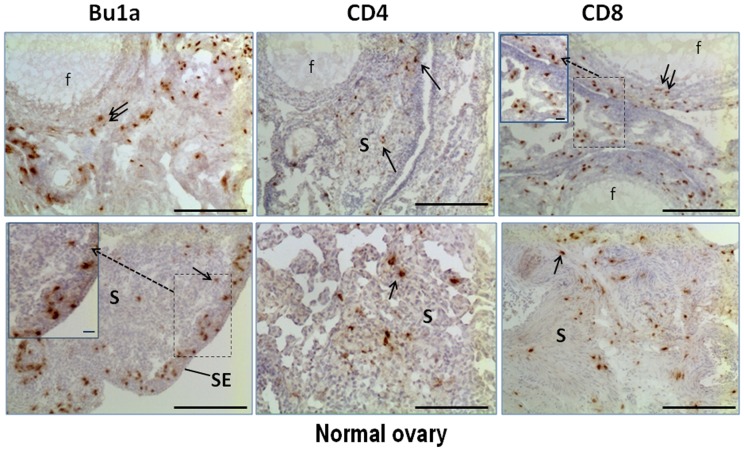
Localization of immune cells in normal hen ovaries. Bu1a+, CD4+ and CD8+ cells (arrows) detected by immunohistochemistry in normal ovaries in the ovarian stroma (S), adjacent to follicles (f), in the thecal layer of follicles (double arrow) and were abundant in the cell layer under the ovarian surface epithelium (SE). Original magnification, 10x; scale bar = 100 µm. Selected areas (dotted boxes) are shown in an inset at higher magnification for Bu1a and CD8 staining; inset scale bars = 10 µm.

**Figure 3:**
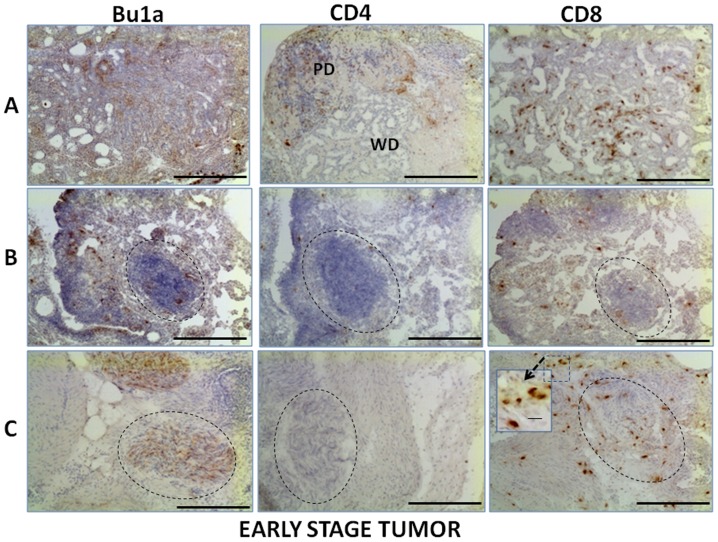
Localization of immune cells in early stage ovarian tumors. Bu1a+, CD4+ and CD8+ cells are shown in similar regions of serial sections that contain tumor foci. More CD8+ T cells and B cells are present in tumor foci than CD4+ T cells. (**A**) Early-stage tumor showing CD4+ cells in a tumor with poorly differentiated (PD) structure, and CD8+ and B cells in an adjacent well differentiated (WD) area. (**B**) A small lesion (dotted circle) containing CD8+ T cells and Bu1a+ cells, but not CD4+ cells. (**C**) An area with neoplastic cells containing CD8+ and Bu1a+ cells, but not CD4+ cells. Original magnification, 10x; scale bar = 100 µm. Stained CD8+ lymphocytes in a selected area (dotted box) is shown in an inset at higher magnification; scale bar = 10 µm.

**Figure 4:**
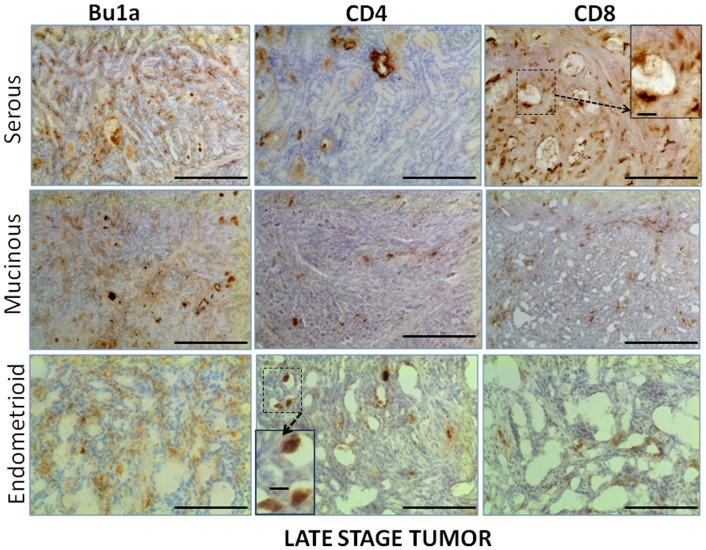
Immune cell distribution in ovaries with late stage ovarian tumors. Similar regions of three tumor types are shown in serial sections stained for Bu1a, CD4 and CD8. *Top Row:* An example of sections from a serous ovarian tumor showing few CD4+ cells within the tumor compared to Bu1a+ and CD8+ cells. *Middle Row:* An example from a mucinous ovarian tumor showing deposits of Bu1a+, CD4+ and CD8+ cells between glands and throughout the tumor. *Bottom Row:* An example of an endometrioid ovarian tumor showing Bu1a+, CD4+ and CD8+ cells. Original magnification, 10x; scale bar = 100 µm. Stained CD8+ and CD4+ lymphocytes in selected areas (dotted boxes) are shown in insets at higher magnification; scale bar = 10 µm.

CD4+ T cells were observed within the thecal layers of follicles in normal ovaries (**Figure 2**) but not in the granulosa cell or oocyte compartments of follicles. CD4+ T cells occurred throughout the normal ovary, occurring sporadically in the stroma and adjacent to follicles, inside atretic follicles (not shown) and near blood vessels. In tumor-containing ovaries, CD4+ T cells occurred in the stroma of both early stage (**Figure 3**) and late stage ovarian tumors (**Figure 4**) and were less frequent than either CD8+ or Bu1a+ cells. In contrast to CD8+ T cells and Bu1a+ B cells, CD4+ T cells were near, but not within, early tumor lesions (**Figure 3**). CD8+ T cells were similarly localized in normal follicles and in the stroma near follicles (**Figure 2**). CD8+ T cells were found in early and late stage tumors (**Figures 3** **and** **4**). While B (Bu1a+) and T cells were similarly distributed in the ovarian stroma, Bu1a+ staining was rarely found in follicles (**Figure 2**).

### Quantitative Assessment of the Number of Immune Cells in Normal Ovary, Early Stage and Late Stage Ovarian Tumors

**Figure 5:**
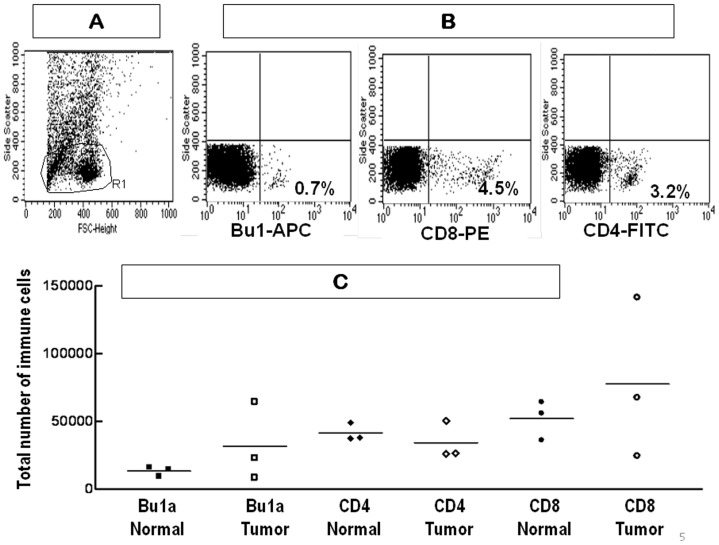
The number of ovarian CD4+, CD8+ and Bu1a+ cells estimated by flow cytometry. (**A**): Representative forward vs. side scatter plot for cells from a whole ovary showing the gate (R1) used for lymphocytes. (**B**) Cells were first gated using the gate in (A) and then stained with either Bu1a-APC, CD4-FITC or CD8-PE. The percent of labeled cells within the gate is shown in the lower right quadrant for each stain. (**C**) The total number of Bu1a-APC, CD4-FITC and CD8-PE labeled cells from normal and tumor hen ovaries (n = 3/group) are shown with the median indicated by the horizontal line.

In initial experiments, the total number of T and B cells isolated from the whole ovary was determined by flow cytometry. A representative flow cytometry scatter plot shows the size distribution of cells from a normal ovary without a tumor (**Figure 5A**) with a gate on the lymphocyte region (R1). The corresponding representative dot plots for each fluorescent label is also shown (**Figure 5B**). Although there appeared to be fewer CD4+ T cells in ovarian tumors compared to normal ovaries (**Figure 5C**), there was also a significant variation in CD4+ cell content among hens, which is likely due to variations in follicle content and tumor stage as seen in cell counts obtained using morphometry. A limitation of flow cytometry is that the whole ovary is used to prepare lymphocytes and thus the location of immune cells relative to tumors or follicles cannot be assessed. Likewise, it was not possible to determine the tumor type, ovarian morphology or tumor stage by histology. The total number of T and B cells was determined further by morphometric evaluation of immunostained sections of normal ovary and ovarian tumors. This approach also made it possible to determine ovarian tumor stage and to assess possible differences in lymphocyte location.

**Figure 6:**
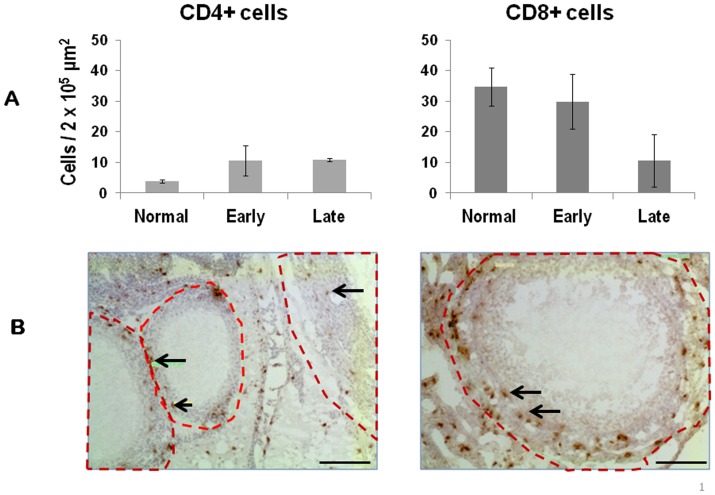
Comparison of the number of immune cells in ovarian follicles determined by morphometric analysis. (**A**) B (Bu1a+) cells were rarely found in follicles and therefore were not counted. The number of CD4+ T cells increased slightly (p = 0.052) and the number of CD8+ T cells decreased (p = 0.009) in late stage ovarian tumors compared to normal ovaries. At each stage there were more CD8+ compared to CD4+ T cells (normal ovary, p = 0.003; early stage tumor, p = 0.008; late stage tumor, p = 0.007). Follicles were defined as shown in (**B**) and cells within the designated area were counted and the average determined per 2×10^5^ µm^2^ area of follicle. Three sections from each ovary for each hen were counted at a magnification of 20x; scale bar = 50 µm. Error bars represent mean ± SEM.

Within follicles (**Figure 6**), CD8+ T cells decreased (p = 0.052) whereas CD4+ T cells increased (p = 0.009) from normal to early tumors and normal to late stage tumors. B cells were not typically associated with follicles and were not counted. The number of follicles decreased or were absent in ovaries with tumors compared to normal ovaries (**Figure 1** **)**.

**Figure 7:**
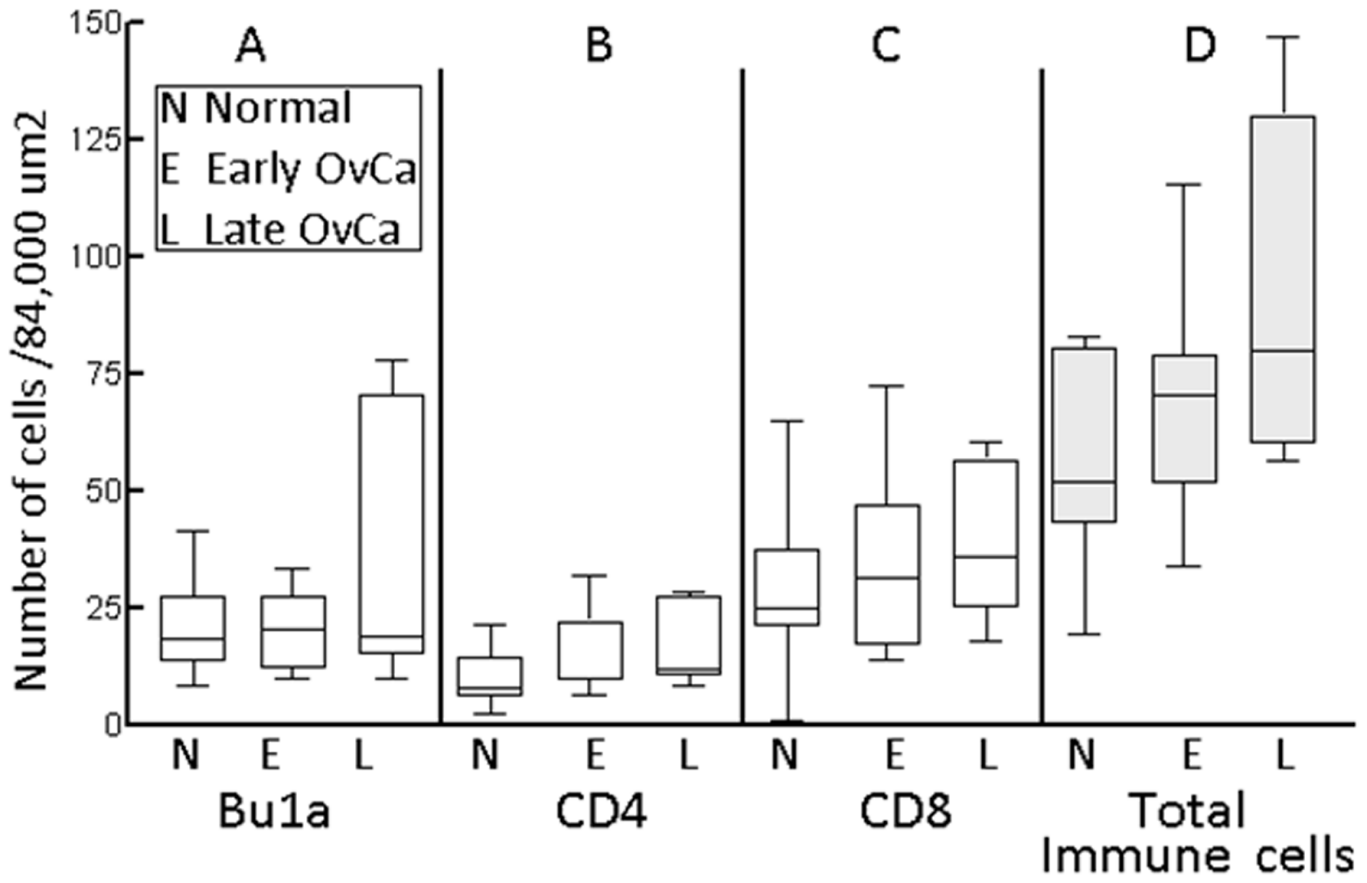
The number of Bu1a+ B cells and CD4+ and CD8+ T cells in normal ovaries and ovarian tumors determined by morphometric analysis. As shown in panels **A-C for normal (N), early (E) and late stage (L) ovarian cancer**, Bu1a+ cells and CD8+ T cells were more numerous overall than CD4+ T cells, particularly in late stage tumors. In panel **D**, the B and T cells counts were added. There was an overall increase in total (non-follicular) immune cells from normal ovary to late stage tumors. Cells were counted in multiple fields in three sections from each ovary for each hen at 20x magnification. The average number of B (Bu1a+) cells and CD4+ and CD8+ T cells was estimated from 11 hens with normal ovaries (number of fields counted was Bu1a = 524, CD4 = 425 and CD8 = 360), 8 hens with early stage ovarian cancer (number of fields counted was Bu1a = 228, CD4 = 190 and CD8 = 225) and 7 hens with late stage ovarian cancer (number of fields counted was Bu1a = 180, CD4 = 190 and CD8 = 200). Since the areas evaluated varied in size, all counts were normalized to 8×10^4^ µm^2^.

The stromal (i.e., non-follicular) immune cell content increased overall with tumor stage (**Figure 7** **)** and was characterized by a higher number of CD8+ T cells than CD4+ T cells at each stage (CD8 vs. CD4 cells for normal ovary, p = 0.003; early stage tumor, p = 0.008 and late stage tumor, p = 0.007). Similar to flow cytometry, Bu1a+ cells and CD8+ T cells were more numerous than CD4+ T cells in normal ovary and early and late stage ovarian tumors (**Figure 7A, B and C**). The number of CD4+ T cells was significantly lower in early stage tumors compared to normal ovary (p = 0.03) and there was no difference between Bu1a+ and CD8+ cells. CD4+ cells were significantly increased (p = 0.05) while the increase in the mean values for Bu1a+ and CD8+ cells did not reach significance in late stage tumors compared to normal. Overall, total immune cells increased from normal to early stage to late stage tumors (**Figure 7D**).

**Figure 8:**
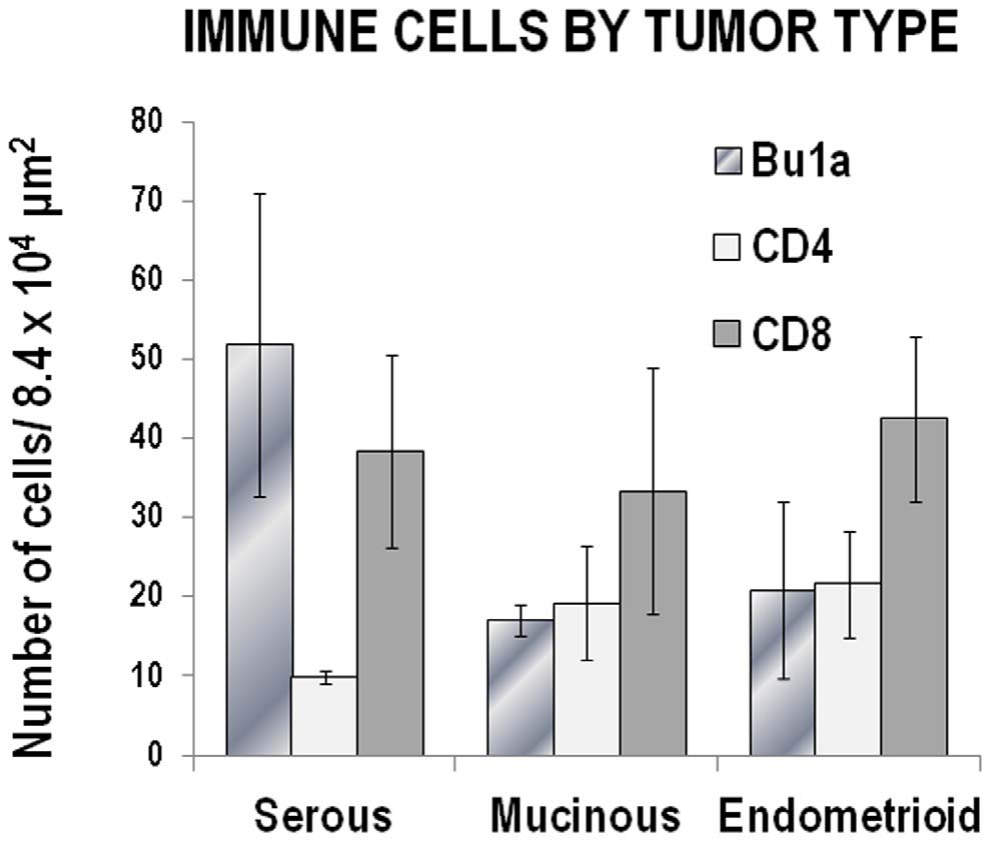
Comparison of the number of Bu1a+, CD4+ and CD8+ cells by ovarian tumor type. Since the histological classification of tumors is clearest in late stage ovarian tumors, the morphometric analysis was confined to late stage tumors. Clear cell tumors occurred in relatively low numbers and were not included in the analysis. The number of Bu1a+, CD4+ and CD8+ cells was counted as described in the Methods for serous (66 fields in 3 hen ovaries); mucinous (53 fields in 2 hen ovaries) and endometrioid (37 fields counted in 2 hen ovaries) histology tumors. There were significantly more B (Bu1a+) cells in serous tumors than in mucinous (p = 2.6×10*^−^* ^14^) or endometrioid (p = 7.6×10*^−^* ^9^) tumors. In contrast, serous tumors had significantly fewer CD4+T cells compared to mucinous (p = 3.8×10*^−^* ^5^) or endometrioid (p = 2.9×10*^−^* ^9^). The number of CD+8 T cells did not differ significantly by tumor histology (p>0.2).

The number of immune cells differed by tumor type (**Figure 8**). Only the late stage (III/IV) tumors were assessed since the determination of tumor histology was clearer at these stages. There were more B cells in serous tumors than in mucinous (p = 2.6×10*^−^* ^14^) or endometrioid (p = 7.6×10*^−^* ^9^) tumors. Serous tumors had significantly less CD4+T cells compared to mucinous (p = 3.8×10*^−^* ^5^) or endometrioid (p = 2.9×10*^−^* ^9^) tumors. The number of CD8+ T cells was similar among tumor types (p>0.2).

## Discussion

This study is the first to investigate immune cells associated with ovarian tumors in the egg-laying hen model of spontaneous ovarian cancer. The total immune cell content in ovaries with tumors was higher compared to that in normal ovaries. The result was similar using two standard methods; flow cytometry and morphometric analysis of immunohistochemically labeled tissue sections. By flow cytometry, there were relatively large variations in cell numbers for each cell type, particularly for the hens with tumors. These variations found by flow cytometry could be partly due to variable inclusion of cells from blood vessels and the variable content of active follicles as the ovary progresses from a normal ovary to ovaries with an advanced stage tumor. Therefore, more emphasis was placed on the determination of immune cells numbers by immunohistochemical morphometry since the location of lymphocytes relative to the tumor or to follicles could be assessed and since tumors could be classified by histology and stage.

The finding of immune cells in the normal ovary in this study is consistent with previous studies in hens [38], other animal models [47] and humans [45], [48]. In a detailed study of the normal rat ovary, lymphocytes were abundant and also increased transiently coincident with ovulation [47]. When rats were splenectomized, ovarian lymphocytes were reduced but not eliminated suggesting that in the normal ovary, there are “resident” lymphocytes, or that not all lymphocytes traffic from the spleen. In this study of the hen, there also appears to be “resident” lymphocytes in the normal ovary which are adjacent to the outer cell layer of the follicles as well as in the ovarian stroma and surface epithelium. This raises a question as to whether immune cells found in ovarian tumors originate from spleen or lymph nodes or whether resident immune cells in the ovary contribute to malignant conversion.

In the normal hen ovary, B cells were found with relatively high frequency in the stroma and medullary regions, similar to CD4+ and CD8+ cells. However, B cells were rarely observed within the follicles, in contrast to CD4+ and CD8+ cells. The relatively high abundance of T cells in the follicles suggests they have a role in normal ovarian function. While the marker Bu1a is specific for hen B cells, it is also expressed on avian macrophages and to a much lesser extent on other non-B cell types [49]. Thus, further study is needed with secondary markers to differentiate B cells and non-B cells in the hen. Nonetheless, this study showed that B cells represent a significant proportion of the lymphocytes in ovarian tumors.

Based on numerous reports from other groups in animal models and humans, immune cells were expected in ovarian tumors [50], [51], [52]. It has been shown that patient survival is correlated with the number of tumor infiltrating lymphocytes [5] in surgical material which usually is obtained from late stage disease. It is difficult to determine the role of these cells in early stages since ovarian cancer is rarely detected early in humans. Likewise when ovarian cancer is induced in animal models it is not clear if the initiation events are models of spontaneous tumor development. There are no studies of immune cells in hen ovarian tumors and few studies that compare these cells in normal ovary and ovarian tumors to assess potential changes during spontaneous ovarian tumor development. In this study we found that the overall number of immune cells in the ovary tends to be higher in the presence of spontaneous tumors, which is consistent with the accepted concept that immune cells traffic to and invade tumors [5], [53], [54]. Furthermore, the immune cell content is highest in late stage tumors, particularly in late stage serous tumors. Since this trend is similar to that in humans [50], [52], [55], [56], [57], the results support the use of the hen model to investigate immune responses to ovarian tumors and for pre-clinical vaccine trials.

In humans, lymphocyte content determined by immunohistochemistry in ovarian tumors is not consistent in different studies, particularly with respect to B cells [58]. Late-stage human ovarian tumor biopsies contained primarily CD8+/CD45RO+ T cells and CD68+ macrophages, while NK and B cells occurred in the lowest numbers [50]. In contrast, CD20+ B cells were found in 42% of ovarian tumors in another study [6]. In survival studies, a higher number of CD19+ B cells was associated with a poorer prognosis [59] while higher numbers of CD20+ B cells were associated with increased disease free survival [6]. Differences in these studies may be due to the use of different markers, but they may also reflect differences in the relative content of different tumor histo-types in these studies [6]. B cells and CD8+ T cells were seen in ovarian tumors of the hen with relatively high frequency, while CD4+ cells were detected at a relatively lower frequency.

Immune cells occurred in all three of the tumor histo-types examined (serous, mucinous, and endometrioid), but there were more immune cells in late stage serous tumors. This is consistent with a recent study of human ovarian tumors using similar markers (CD3, CD4, CD8, and CD20) which showed that tumor infiltrating lymphocytes were more prevalent in high-grade serous carcinomas followed by endometrioid ovarian tumors and then other ovarian tumor types [6]. Also, similar to human ovarian tumors [6], the relative content of B cells compared to T cells was higher in serous than other histo-types in the hen. This suggests several possibilities: the immune response may differ among different tumor types or the influence of the tumor on regulation of immunity by the different tumor types differs.

The results of numerous investigations have shown many similarities between ovarian cancer in hens and humans [8], [60]. There are minor physiologic differences between hen and human ovaries [61]. One difference is that birds have a unilateral ovary at maturity while mammals have bilateral ovaries. Another difference is the absence of a post-ovulatory luteal phase in avian ovulatory cycles. In mammals, the eggs are released during ovulationand if fertilitzed, implant in the uterus. Cells remaining in the follicle form a corpus luteum, a temporary endocrine gland which produces relatively high levels of progesterone and moderate levels of estradiol and inhibin to maintain pregnancy. Since hens deposit eggs externally there is no need to maintain the implanted embryo and thus the follicle regresses. It is not clear if these minor differences in the ovary are important to the use of the hen model to study development of ovarian tumors.

Immunity in hens and mammals are comparable although with minor differences [62], [63]. For example, nearly all hen MHC genes have counterparts in the human MHC. The MHC region in chickens is simpler and more compact [64], [65]. Also, the immunoglobulin system of chickens differs from humans; chickens have structural and functional equivalents of mammalian IgM, IgA, and IgG, although homologs of IgE or IgD have not been found [66]. Similar to all higher vertebrates, primary diversity in chicken VH regions is created by V-D-J recombination, combined with somatic hypermutation [67]. However, there are other mechanisms of VH diversification in the chicken that differ from those in mice and humans [67], [68].

In summary, the strength of this spontaneous animal model of ovarian cancer is the similarity with human ovarian cancer. This study is unique since it describes the immune cell content and location in early to advanced stage ovarian tumors of hens. The results provide a foundation for future delineation of immune cells (e.g., regulatory T cells, macrophages) and markers of immune cell activation using the hen model. Use of this model will make it possible to examine the relationship of immunity to the transition from normal ovary to early stages of ovarian tumors, studies which are virtually impossible in human ovarian cancer.
